# Eating Speed and Its Associations with Cardiometabolic Risk Factors in Children

**DOI:** 10.3390/children12121686

**Published:** 2025-12-11

**Authors:** Manuel Abraham Gómez-Martínez, Diana Rodríguez-Vera, Gabriela Olivares Mendoza, Fernanda Lobato Lastiri, José A. Morales-González, Rodolfo Pinto-Almazán, Arely Vergara-Castañeda

**Affiliations:** 1 Promotion and Education for Health and Food Research Group, Universidad La Salle Mexico, Benjamin Franklin 45, Colonia Condesa, Mexico City 06140, Mexico; mgomez@facmed.unam.mx (M.A.G.-M.);; 2 Public Health Department, Faculty of Medicine, National University of Mexico, Escolar 411A, Copilco Universidad, Coyoacán, Mexico City 04360, Mexico; 3 Sección de Estudios de Posgrado e Investigación, Escuela Superior de Medicina del Instituto Politécnico Nacional, Plan de San Luis y Diaz Mirón s/n, Mexico City 11340, Mexico; nutriologadianavera@gmail.com (D.R.-V.);; 4 Clínica de Nutrición Especializada Béke, Colonia San Sebastián, Texcoco 56130, Mexico; 5 Programa de Maestría en Ciencia de los Alimentos y Nutrición Humana, Facultad de Ciencias Químicas, Universidad La Salle México, Mexico City 06140, Mexico; 6 Laboratorio de Medicina de Conservación, Escuela Superior de Medicina del Instituto Politécnico Nacional, Plan de San Luis y Diaz Mirón s/n, Mexico City 11340, Mexico; jmorales101@yahoo.com.mx; 7 Fundación Vithas, Grupo Hospitalario Vithas, 28043 Madrid, Spain

**Keywords:** eating behaviors, chronic diseases, cardiovascular risk factors, lipid profile, school-aged children

## Abstract

**Highlights:**

**What are the main findings?**

- Fast eating was linked to a more adverse cardiometabolic profile (higher BMI and waist circumference, elevated diastolic blood pressure, triglycerides, and total cholesterol, alongside lower HDL) relative to slower eating.
- In this cohort of Mexican schoolchildren (6–12 y), 17.7% were fast eaters; a male preponderance was observed but was not statistically significant (*p* = 0.10).

**What are the implications of the main findings?**

- Self-reported eating speed should be incorporated into pediatric cardiovascular risk appraisal in schools and primary care as a low-burden behavioral marker.
- Slowing eating may be a target for primary prevention; rigorously designed longitudinal and intervention studies are warranted to establish causality and quantify risk reduction.

**Abstract:**

**Background/Objective:** Mexico has experienced an increase in the prevalence of overweight and obesity among schoolchildren, predisposing them to type 2 diabetes mellitus. In addition, rapid eating has been increasingly implicated in the dysregulation of appetite control, greater energy intake, and adverse metabolic outcomes in children. Prior evidence indicates that a faster eating pace is associated with excess adiposity and lipid metabolism. This study aimed to compare cardiovascular risk factors (waist circumference, waist-to-height ratio, body mass index (BMI), and lipid profile) among school-aged Mexican children according to self-reported eating speed. **Design:** Cross-sectional observational study. **Setting:** Public elementary schools in Mexico. **Participants:** Ninety school-aged children (52.2% female) aged 6–12 years old. Eating speed was assessed using an adapted and validated self-administered questionnaire. **Intervention:** No intervention was applied; participants were classified into slow-, normal-, or fast-eating groups according to their usual eating speed as reported in the instrument, which includes questions regarding self-perception and family perception. **Main Outcome Measure:** The primary outcomes included anthropometric parameters (BMI, waist circumference, and waist-to-height ratio), blood pressure (systolic and diastolic), and biochemical markers of lipid metabolism (triglycerides, total cholesterol, and HDL cholesterol). **Analysis:** Descriptive statistics were computed, and comparisons across eating speed groups were performed using one-way ANOVA for continuous variables and chi-square tests for categorical data. Statistical significance was set at *p* < 0.05. **Results:** Among the 90 children evaluated, 17.7% were classified as fast eaters. Although gender differences in eating speed were not statistically significant (χ^2^= 4.607, *p* = 0.100), a higher proportion of boys were classified as fast eaters. Children in the fast-eating group exhibited significantly higher BMI (1.4 kg/m^2^), waist circumference (4 cm greater), and modest elevations in triglyceride and total cholesterol levels, alongside lower HDL cholesterol, relative to their slow-eating peers (all *p* < 0.05). Among all variables, only diastolic blood pressure differed significantly across groups (F = 3.92, *p* = 0.022), with fast eaters showing the highest values. Nevertheless BMI, waist circumference, triglyceride levels, and total cholesterol were not statistically significant in the logistic regression, and HDL cholesterol demonstrated an association close to 95% [0.051 (0.011–0.226)] to a protective factor against cardiometabolic events, estimating an effect size of 1.64 using Cohen’s d, which is considered a large effect, when compared to their slower-eating peers. **Conclusions and Implications:** Faster eating speed was consistently associated with unfavorable anthropometric and lipid profile indicators, aligning with previous evidence linking rapid eating to early cardiometabolic alterations. These findings emphasize the relevance of including eating behavior assessments in pediatric cardiovascular risk screenings and prevention strategies.

## 1. Introduction

Over the past two decades, the prevalence of childhood obesity among Mexicans has risen at an alarming rate, and it is a worldwide problem [1]. National surveys indicate that the combined prevalence of overweight and obesity in Mexican children aged 5–11 years has increased from 26% in 1999 to nearly 38% in 2021, reflecting an epidemiologically significant upward trend [2]. Furthermore, this is particularly concerning given the early onset of abdominal adiposity and its association with a considerable number of cases of type 2 diabetes mellitus, dyslipidemia, hypertension, and metabolic syndrome in children and adolescents [3,4,5], and the fact of its influence during early stages of life on conditions in adulthood [6].

Excess weight in childhood is strongly linked with different risk factors considered behavioral determinants, such as lack of physical activity, long commutes from home to schools, and an unhealthy diet that may be influenced by family income, socioeconomic status, and geographic residence location, which can affect the affordability of certain foods. Some of these factors can determine the amount of food that is consumed, which could lead to a large quantity of food in one meal [7].

Among the behavioral determinants of childhood adiposity, eating behaviors have received growing attention. Satiety responsiveness, eating until full, and eating speed are concepts that describe interrelated aspects of eating patterns [8,9], in which several physiological and behavioral mechanisms have been proposed to explain how eating speed influences adiposity and metabolic risk. Rapid eating is associated with attenuated cephalic-phase responses, delayed secretion of anorexigenic hormones such as peptide YY and glucagon-like peptide-1, and reduced perceived fullness. This implies that eating quickly can lead to consuming more food and delaying the feeling of satiety, which creates a false perception of having eaten enough and puts individuals at risk of increased calorie intake and weight gain, as well as higher and faster glucose spikes, which increase the demand for insulin and promote long-term resistance [9,10,11].

Similarly, some other reports have shown that eating swiftly is an important determinant for some adverse metabolic outcomes, such as high levels of LDL-c and TGL, inverse to low levels of HDL-c and high fasting plasma glucose [12,13]. Interestingly, a fast rate of eating was associated with the previously mentioned serum components after adjustment for BMI, suggesting that other independent pathways may operate for these factors [14,15]. This is likely attributable to excessive energy intake and reduced fat oxidation due to insufficient physical activity. Furthermore, accelerated ingestion can induce autonomic stress by activating the sympathetic nervous system, resulting in increased blood pressure and heart rate, thereby heightening cardiovascular risk.

Finally, the rapid intake of excess calories has been linked to systemic inflammation, specifically to increased production of pro-inflammatory cytokines, which are key in the development of atherosclerosis [16].

Notwithstanding, it is difficult to determine when a rapid pace of eating speed becomes an established eating habit; a person who exhibits rapid eating behavior during childhood is likely to persist into adulthood, emphasizing the need to identify behaviors that contribute to excessive weight gain at early development stages [17,18].

While numerous studies in adults have linked fast eating with increased BMI, metabolic syndrome components, and dyslipidemia, the primary interest in the Mexican pediatric population targeted in this study stems from the lack of sufficient studies focusing on the relationship between eating speed and abdominal obesity or other cardiovascular and metabolic risks among children. For this reason, this study aimed to compare cardiovascular and metabolic risk factors—such as waist circumference, waist-to-height ratio, body mass index, and lipid profile—among children according to their self-reported eating speed based on a validated questionnaire.

## 2. Materials and Methods

### 2.1. Study Design and Population

A cross-sectional study was performed; participants were recruited from a public elementary school in Mexico City. The sample size was 90 children with non-probabilistic enrolment, both boys and girls aged from 9 to 11 years old. Exclusion criteria included thyroid disease, any other previously diagnosed chronic illness that might cause weight change, eating disorders, and the consumption of pharmacological treatment that might alter appetite or dieting.

The sample size of 90 children was determined by logistical feasibility and aligns with other exploratory studies in pediatric populations. Considering the prevalence data of fast eating habits among school-aged children (~15–20%) and a desired confidence level of 95% with an allowable margin of error of 10%, a minimum sample size of 81 was estimated. We recruited 90 children to ensure sufficient statistical power and enable subgroup analyses.

Participants were selected via non-probabilistic convenience sampling from public elementary schools. Before they participated in the study, informed consent was obtained from the parent or guardian of each child, and the children also signed a letter of agreement. The Medical Ethics Committee of the Mexican Medicine Faculty at the Salle University approved the study (Q-097/13).

### 2.2. Data Collection

Anthropometric and clinical measurements: All evaluations were performed by a trained team according to the anthropometry standardization manual and the AHA (American Heart Association) global hypertension practice guidelines [19,20] for blood pressure. Weight and height were measured without shoes and excess clothing removed. Weight was taken using a digital scale (FitScan BC-601F, Tanita^®,^ Tokyo, Japan) to the nearest 0.1 kg; height was measured using a stadiometer (SECA 213^®^, Germany Waist circumference was measured at the end of an exhalation with a non-elastic flexible tape (metal tape measure, Rosscraft ®, Washington, DC, USA), in the standing position at the level of the midpoint between the lower costal border and the iliac crest. BMI was calculated as the body weight (kg) divided by the square of the height (m^2^). Also, the waist-to-height ratio was computed and categorized using a previous report in the Mexican population [5]. Systolic blood pressure (SBP) and diastolic blood pressure (DBP) were measured using a Welch Allyn sphygmomanometer (DS44-MC DuraShock DS44 sphygmomanometer Welch Allyn ®, Skaneateles Falls, NY, USA) with a specific child’s bracelet. The diagnosis of hypertension was based on age- and gender-specific percentiles described elsewhere [18]; meanwhile, overweight and obesity were defined according to the International Obesity Task Force’s references for children [21].

Biochemical analyses: Fasting blood samples were obtained from all subjects, and triglycerides (TGL), high-density lipoprotein cholesterol (HDL-c), and total cholesterol (TC) were determined in an autoanalyzer, Mindray BS-200^®^. Low-density lipoprotein (LDL) cholesterol was estimated indirectly with the Firedewald equation ([LDL cholesterol] = [total cholesterol] − [HDL cholesterol] − [triacylglycerol]/5) used in most clinical laboratories [22]. For dyslipidemia, we considered higher limits (lower in the case of HDL-c) recommended by the American Academy of Pediatrics and the National Cholesterol Education Program of the United States of America; TC > 200 mg/dL, LDL > 130 mg/dL, HDL < 35 mg/dL, and TGL > 150 mg/dL [18]. All assays were performed in strict compliance with institutional protocols, utilizing standardized reagent kits, and the institution’s laboratory division meticulously collated the resultant data.

Dietary assessment: Dietary intake was assessed by a standardized dietitian using the multiple-pass 24 h recall (this questionnaire was validated in the Mexican population, showing highly significant correlations (between 0.69 and 0.93) in almost all variables). The average differences between means ranged from −14% to 10.9%. No differences were found in total servings by energy density [23]. On the other hand, there are no validation studies in the target population. Data were analyzed using the Food Processor ESHA research software (version 11.9.x), which includes nutritional values for Mexican items.

While the eating behavior questionnaire was adapted from previously published tools, it has not been formally validated in children. Its application in this context should be interpreted as exploratory, pending further psychometric validation studies in pediatric populations.

Questionnaire: A self-administered questionnaire regarding eating behaviors was distributed to each subject, and a trained person oversaw the completion by the children. This questionnaire was composed for the following aspects: eating behavior about eating speed associated with other activities that the individual performs while eating, if they eat until they are satisfied, and if they always eat at the same time. Information concerning eating speed was based on 3 items, as follows: The first question was “Generally, when you eat with other people?”, and the three possible responses were semi-quantitative categories: *“I’m the first finishing my meal”, “I finish my food at the same time as them”, or “I’m always the last”.* To corroborate this item, the questionnaire also included the next questions: “How fast do you eat?” with three possible responses *“fast”, “normal”, “slow”,* and the last one: “Sometimes they’ve told me… ?” and the three possible responses were *“I eat fast”, “I eat slow” or “They’ve never told me that I eat too fast or too slow”.* This last question was used for analyses and comparisons, as it is like the assessment of eating behavior in other reports.

*According to these responses, they were allocated into one of three groups: “fast eating”, “normal eating”, “and slow eating”.*

### 2.3. Data Analysis

All returned questionnaires were double-checked for accuracy and consistency. The collected data were coded, and analyses were performed using the SPSS statistical package for Windows version 22.0 (IBM SPSS Statistics^®^, Armonk, NY, USA). Participants were classified into three eating-speed categories, fast, normal, and slow eating, and comparisons for numerical variables between groups were performed by an ANOVA test. The differences in the proportion of answers for categorical variables across the rate of eating categories were assessed by a chi-squared test. Odds ratios were estimated using binary logistic regression, treating eating speed as the exposure factor. All reported *p*-values were two-sided, and a *p*-value < 0.05 was considered statistically significant.

## 3. Results

### 3.1. Demographic Results

A total of 90 children were included; the mean age of participants was 10.3 ± 1.19 years, and 52.2% were girls. A total of 17.7% of all the children reported fast eating. The characteristics of the study participants according to their eating speed are shown in Table 1.

### 3.2. Associations Between Eating Speed and Cardiovascular Risk Factors

Children who reported eating faster have a higher BMI, a larger waist circumference, higher levels of triglycerides and total cholesterol, and lower HDL compared with those who reported a normal or slow eating speed, without statistical significance. Additionally, there were no differences according to gender, despite a higher proportion of boys who reported fast eating compared with girls (68.7% vs. 31.2%, respectively; *X*^2^ = 4.607; *p* = 0.100). Also, we found a statistical tendency for fast speed according to their BMI classification (*X*^2^ = 11.928; *p* = 0.064).

On the other hand, dietary intake was compared among groups according to eating speed, and no differences were found in total energy intake and macronutrients (grams or percentages).

**Figure 1:**
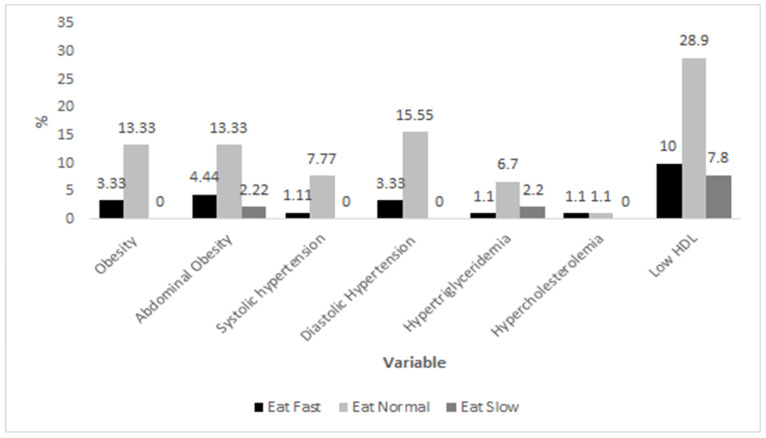
Prevalence of cardiovascular risk factors according to eating speed in children. Prevalence (%) of selected cardiovascular risk factors stratified by eating speed category (fast, normal, slow). Data are based on study participants’ clinical and biochemical measurements.

Regarding cardiovascular risk factors, the most common alterations across all children were low HDL cholesterol, abdominal obesity, and hypertriglyceridemia (46.7%, 20%, and 10%, respectively), whereas those with lower prevalence were hypercholesterolemia and high LDL cholesterol, with 2.2% in both cases. Figure 1 shows the prevalence of cardiovascular risk factors according to their speed of eating, where there seems to be a higher percentage of children with obesity, diastolic hypertension, and low HDL cholesterol who reported eating normally. Despite this trend, estimated odds ratios showed no association between those who reported fast eating in comparison with normal and low eating (Table 2).

## 4. Discussion

It is known that eating speed is acquired as an eating habit, and it has been suggested that a person who has a rapid eating behavior during childhood is likely to retain it into adulthood [13].

Although there are standard instruments to assess dietary intake, such as food frequency questionnaires or 24 h dietary recalls typically administered by trained professionals, self-reported measures remain widely used. Self-reported eating speed has been consistently validated in previous studies against more direct, objective observational measures (such as mealtime duration recorded by video or food weight consumed per unit of time). For instance, individuals identifying as “fast eaters” reliably demonstrate a significantly higher eating rate with some other objective measures, or the information provided by a friend or others [24,25,26], and it has been considered a true reflection of eating behaviors in daily life [14]. The inclusion of this measure is both practical and scalable for large-scale epidemiological studies, allowing to capture a robust proxy marker of the individual’s habitual perception and chronic behavioral pattern, which is the most relevant factor for the long-term development of cardiometabolic risk factors.

In this research, we found a global prevalence of fast eating (12.7%) that falls within the range previously reported (7.2–21.3%) [8,20]. Regarding gender, the highest prevalence was reported in boys, although non-statistical differences were found. In concordance, Ochiai et al. previously reported similar results, yielding a prevalence of around 21.7% in boys [8]. Several reports suggest that men eat faster than women because women take more bites and a longer time to eat than men [8,27].

Although BMI is the gold standard for classifying the nutritional status of children and adults, including overweight or obese individuals [26], many studies have linked eating quickly with higher weight, BMI [28,29], and waist circumference [13]. Reports indicate that the relationship between eating speed and being overweight is related to total energy intake [12,30,31]. In this sense, a meta-analysis that included 22 studies that experimentally manipulated eating speed reported that slower eating led to a reduction in food intake, in comparison with those with fast eating, which reported an increased food intake [9]. For this reason, it has been proposed that slowing the rate of ingestion allows more time for the processes themselves and lengthens the satiety curve, reducing total energy intake [32]. In other words, eating swiftly leads to a “satiety unresponsiveness” where the individual fails to respond to normal satiety signals, such as gastric distension, whereas eating at a moderate rate of speed results in a more pronounced anorexigenic gut peptide release and response than eating very fast [33,34,35].

We focused our analysis on demonstrating whether eating behaviors could influence biochemical determinations such as lipid and glucose profiles, as well as in previous reports, in which Sakurai M et al. associated the incidence of diabetes mellitus type II, through the body weight gain effect, in persons who eat fast [30], and some other reports have found positive associations between eating rate and insulin resistance [24,36,37].

Several studies have shown a trend of association between eating fast food with higher BMI, waist circumference, diastolic blood pressure, triglycerides, total cholesterol, and LDL-cholesterol levels [38]. In concordance, our results are similar to those reports among adults and children, which suggest that an increased speed of eating could be associated with an increased risk of obesity [39,40,41] and with a higher probability of developing a metabolic disorder, high blood pressure, hyperglycemia, and abnormal lipid profile in boys than girls, independently to the weight or BMI as reported in some other reports [42,43] where these associations remained statistically significant even after adjusting for weight or BMI, especially for low HDL cholesterol as our results pointed out [38,40].

While less precise, BMI remains a fundamental and highly reliable screening tool for general adiposity and is indispensable for classifying overweight and obesity in the clinical setting. Moreover, the use of WC, and by extension the WHtR, is considered a predictor of metabolic risk in children, and they are not merely acknowledged, but highly supported by international guidelines. Therefore, the combination of these indicators provides a robust, comprehensive picture of overall and central adiposity, maximizing the assessment of risk.

Despite the mechanisms by which eating rate influences metabolism have not been fully elucidated. In our findings, there is a higher proportion of high WC in children who eat fast. This result is important because Klünder et al. reported that WC is more sensitive than BMI in predicting metabolic or cardiovascular diseases [5]. The authors found that excess abdominal fat promotes the increase in free fatty acids and proinflammatory cytokines, related to the development of insulin resistance, dyslipidemia, and high blood pressure [40,44].

The observed trends in this study may be a starting point for the development of more complete studies that analyze in greater depth the relationship between the speed of eating and metabolic disorders in the Mexican population.

Among the limitations, we acknowledge that although this study included three methods to assess children’s eating speed, only the question ‘How fast do you eat?’ was analyzed, considering that the other ones are not comparable with other similar studies that have made this assessment.

Another disadvantage of the present study is the small sample size. Nevertheless, the effect size of approximately 1.64 indicates that the observed effect is unlikely to be random. For this reason, we suggest enlarging the number of subjects and performing another accurate measurement, such as recording eating time and assessing diet, as well as objective measures of satiety response, to demonstrate a higher intake in those who eat quickly and control the association according to total energy intake in a large follow-up, adjusting for energy intake or activity in multivariable models.

Although dietary intake was recorded, detailed group-specific comparisons of energy and macronutrient intake were not presented. These should be included in future analyses to enhance clarity and transparency.

Despite the fact that physical activity is a well-established determinant of cardiovascular and metabolic health, it was not quantitatively assessed in this study. Future protocols should include validated tools such as the Physical Activity Questionnaire for Children (PAQ-C) to control for its confounding effects.

Strengths of this study include the preliminary findings of cardiometabolic risk factors and behavioral determinations, because there are no reports on this field in the Mexican children’s population. Future studies should explore these hypotheses in a large sample and across different age ranges. These results underscore the potential of behavioral interventions aimed at reducing eating speed as a viable component of comprehensive weight management and metabolic health improvement programs [45].

Though statistical significance was not reached, the observed trends propose that faster eating speed may be linked to less favorable anthropometric and lipid profiles in school-aged children. Importantly, the clinical relevance of these findings, including a 1.4 kg/m^2^ higher BMI and elevated triglycerides in the fast-eating group, warrants further study. Larger, multi-centered investigations incorporating physical activity data and more robust dietary assessments are needed to confirm these relationships.

## 5. Conclusions

The burgeoning epidemic of childhood obesity in Mexico over the last decade and a half signals an alarming public health crisis with far-reaching implications for future generations.

This study’s comprehensive exploration into the nexus between eating speed, obesity, and associated cardiovascular risk factors among Mexican school-aged children sheds light on the intricate interplay of dietary behaviors and metabolic health. The findings underscore a disturbing trend: rapid eating behaviors not only predispose children to increased BMI and waist circumference but also elevate triglycerides and total cholesterol levels and diminish HDL-c levels, marking a clear path towards cardiovascular morbidity.

This analysis elucidates that the velocity of food consumption is not merely a habit acquired in isolation but a lifestyle pattern that likely persists into adulthood, with profound implications for metabolic syndrome components. The study’s findings reveal that rapid eating remains a significant determinant for metabolic dysregulation, independent of BMI adjustments, suggesting alternative mechanistic pathways that warrant deeper investigation. This is particularly relevant in the context of abdominal obesity’s role as an independent harbinger of type 2 diabetes mellitus, dyslipidemia, hypertension, and metabolic syndrome in the pediatric demographic, with the propensity to extend these ailments into adult life.

In dissecting the behavioral aspect of eating speed, this research contributes to a nuanced understanding that transcends traditional nutritional studies. Correlating fast eating with not just an increased propensity for obesity but also with specific biochemical markers of cardiovascular risk, it highlights the critical need for integrating behavioral interventions into obesity prevention and management frameworks. The study’s methodological approach, encompassing anthropometric measurements, clinical assessments, and dietary intake analysis, underscores the multifactorial nature of obesity and cardiovascular risk, advocating for a holistic strategy in tackling these issues.

The findings from this study serve as a clarion call for public health initiatives focused on moderating eating behaviors as a viable lever for curtailing the obesity epidemic among children. Given the natural complexities of metabolic health, it becomes imperative to foster dietary habits that promote satiety and metabolic balance, thereby mitigating the risk of chronic diseases. Future research endeavors must aim to unravel the biological underpinnings that link rapid eating to metabolic dysregulation, providing a scaffold for targeted interventions.

This investigation not only augments the corpus of knowledge surrounding pediatric obesity and cardiovascular risk but also highlights the transformative potential of modifying eating speed in altering the trajectory of metabolic health among children.

## Figures and Tables

**Table 1:** Characteristics of study participants according to their eating speed.

Variables	Total N = 90	Fast Eating n = 16	Normal Eating n = 58	Slow Eating n = 16	*p*-Value
Age, years	10.03 ± 1.19	10.06 ± 0.97	10.03 ± 1.27	10.01 ± 1.17	0.993
Weight, kg	36.64 ± 11.29	40.91 ± 12.46	36.42 ± 11.25	33.18 ± 9.39	0.149
Height, cm	137.73 ± 9.89	140.46 ± 7.85	137.42 ± 9.96	136.13 ± 11.43	0.43
WC, cm	66.53 ± 11.76	70.73 ± 12.28	66.70 ± 11.88	61.73 ± 9.46	0.09
BMI, kg/m^2^	19.01 ± 4.08	20.39 ± 4.34	18.99 ± 4.15	17.06 ± 3.20	0.169
WtHR	0.48 ± 0.07	0.50 ± 0.08	0.48 ± 0.07	0.45 ± 0.05	0.15
SBP, mmHg	100.03 ± 9.99	101.85 ± 9.11	100.81 ± 10.47	95.41 ± 7.95	0.11
DBP, mmHg	68.80 ± 7.38	70.77 ± 6.29	69.31 ± 7.57	64.97 ± 6.71	0.05 ^a,^*
Triglycerides, mg/dL	89.38 ± 48.43	96.37 ± 77.97	88.87 ± 41.25	84.25 ± 43.75	0.77
Cholesterol Total, mg/dL	133.3 ± 34.29	139.93 ± 34.32	133.10 ± 34.28	127.56 ± 35.33	0.59
HDL cholesterol, mg/dL	38.30 ± 17.01	36.12 ± 13.85	38.48 ± 17.03	39.81 ± 20.33	0.82
LDL-cholesterol, mg/dL	77.15 ± 25.46	84.53 ± 23.39	76.84 ± 26.86	70.90 ± 21.34	0.31

BMI: Body Mass Index; WC: Waist circumference; WtHR: Waist-to-Height Ratio; SBP: Systolic Blood Pressure; DBP: Diastolic Blood Pressure. ^a^ Post hoc difference between the fast-eating group vs. the slow-eating group * *p* < 0.05 ANOVA.

**Table 2:** Odds ratio estimations for fast eating speed and cardiovascular risk factors.

Cardiovascular Risk Factor	OR, IC (95%)
Overweight/Obesity	2.19 (0.68–6.97)
High waist circumference	1.74 (0.468–6.273)
Abdominal obesity	1.35 (0.260–7.283)
High systolic blood pressure	0.74 (0.083–6.50)
High diastolic blood pressure	0.99 (0.24–3.975)
Low HDL cholesterol	0.051 (0.011–0.226)
Hypertriglyceridemia	1.167 (0.122–11.194)
Hypercholesterolemia	4.86 (0.288–8.222)

All ORs were estimated considering normal and low eating speed as the reference group.

## Data Availability

The original contributions presented in this study are included in the article. Further inquiries can be directed to the corresponding author.

## Abbreviations

The following abbreviations are used in this manuscript:

AHA	American Heart Association
BMI	Body Mass Index
DBP	Diastolic Blood Pressure
GLP-1	Glucagon-Like Peptide-1
HDL-c	High-density lipoprotein cholesterol
LDL	Low-density lipoprotein
PAQ-C	Physical Activity Questionnaire for Children
PYY	Peptide YY
SBP	Systolic Blood Pressure
TC	Total Cholesterol
TGL	Triglycerides
WC	Waist circumference
WtHR	Waist-to-height ratio
